# Impact of *IL1R1* and *IL1R2* gene polymorphisms on risk of osteonecrosis of the femoral head from a case–control study

**DOI:** 10.1002/mgg3.557

**Published:** 2019-01-08

**Authors:** Feimeng An, Jiaqi Wang, Hongyan Gao, Chang Liu, Ye Tian, Tianbo Jin, Wanlin Liu, Jianzhong Wang

**Affiliations:** ^1^ Inner Mongolia Medical University Hohhot Inner Mongolia China; ^2^ Department of Trauma Orthopedics the Second Affiliated Hospital of Inner Mongolia Medical University Hohhot Inner Mongolia China; ^3^ Key Laboratory of Resource Biology and Biotechnology in Western China (Northwest University) Ministry of Education, School of Life Sciences, Northwest University Xi'an Shaanxi China

**Keywords:** case–control study, Chinese Han population, genetic polymorphism, *IL1R1*, *IL1R2*, ONFH

## Abstract

**Aim:**

Osteonecrosis of the femoral head (ONFH) refers to bony changes caused by osteocyte death under the effects of complicated factors, which is caused by genetic factors and certain risk factors. Our study aimed to explore whether *IL1R1/IL1R2* polymorphisms influenced ONFH risk in the Chinese Han population.

**Methods:**

We selected 286 patients and 441 controls, with 11 single‐nucleotide polymorphisms in *IL1R1* and *IL1R2* gene were successfully genotyped, and evaluated the associations using the chi‐squared test, Fisher's exact test, *T* test, and genetic model analyses. Odds ratios and 95% confidence intervals (CIs) were calculated using unconditional logistic regression.

**Results:**

In the allele model, rs11674595 in *IL1R2* was associated with increasing the risk of ONFH, the rs10490571 and rs3917225 in *IL1R1* gene were associated with an increased risk of ONFH, respectively. In the genetic model, the rs11674595 in *IL1R2* gene was associated with an increased risk of ONFH in the codominant model, dominant model, and log‐additive model, respectively. The rs10490571 and rs3917225 in *IL1R1* gene conferred an increased risk of ONFH in the codominant model, dominant model, and log‐additive model, respectively. We found none of the haplotypes in the *IL1R2* gene was significantly associated with theONFH risk.

**Conclusion:**

Our findings have demonstrated that the rs11674595 (*IL1R2*), rs10490571, and rs3917225 (*IL1R1*) were significantly associated with increasing the ONFH risk in the Chinese Han population.

## 1 INTRODUCTION

Nontraumatic osteonecrosis of the femoral head (ONFH), known as avascular necrosis, is a refractory and progressive disease and caused by osteocyte death (Mankin, 1992; Yu et al., 2016). However, the specific pathogenesis of ONFH has not completely stated. ONFH is believed to be a multifactorial disease that is associated in some cases with both genetic factors and certain risk factors (Björkman et al., 2004; Du et al., 2016; Su et al., 2016). These risk factors include corticosteroid use, alcohol intake, smoking, and various chronic diseases (renal disease, hematological disease, inflammatory bowel disease (IBD), postorgan transplantation, and hypertension) (Zheng et al., 2014).

Previous study has reported that the immune system is substantially involved in the regulation of bone homeostasis;furthermore, ONFH may be caused by disruption of the immune system via lipopolysaccharide activated toll‐like receptor 4 (*TLR4*) signaling (Okazaki et al., 2009). Therefore, abnormal immune responses may contribute to the pathogenesis of ONFH by impacting bone remodeling. Interleukin (IL)‐1 is a primary proinflammatory cytokine, and it could stimulate the expression of genes that associated with inflammation and immunity (Strand & Kavanaugh, 2004). Previous studies have also indicated the association between immune‐related genes and various bone disease. For example, the *IL‐1* gene cluster was related to increase the risk of ankylosing spondylitis (Maksymowych et al., 2006); inhibiting the IL‐1 could reduce cartilage damage in rheumatoid arthritis (Strand & Kavanaugh, 2004); and the recently discovered *IL‐33* as an *IL‐1* cytokine family member has been proved to be specifically released from osteonecrotic bones (Saidi et al., 2011).

Interleukin‐1 receptor, type 1 (*IL1R1*; OMIM: 147810) and IL‐1 receptor, type 2 (*IL1R2*; OMIM: 147811) are cytokine receptor that belongs to the *IL‐1* receptor family, which is an important mediator involving in many cytokines induced by immune and inflammatory responses (Sims & Dower, 1994). Study showed that *IL1R1* and *IL1R2* genes could regulate the cell metabolism, and the response of immune inflammatory induced by many cytokines (Dinarello, 1994; Rock, Hardiman, Timans, Kastelein, & Bazan, 1998). Moreover, the epidemiological studies have been manifested that ONFH was impacted by hereditary factors. Therefore, the *IL1R1* and *IL1R2* genes may be associated with ONFH.

To identify the associations between ONFH and susceptibility loci in previous studies, we conduct a case–control study and identify the relationship between ONFH and the susceptible single‐nucleotide polymorphisms (SNPs) in the *IL1R1* and *IL1R2* gene to further clarify their potential roles in ONFH risk in the Chinese population.

## 2 MATERIALS AND METHODS

### 2.1 Ethics statement

This investigation was conducted in accordance with the ethical standards of the Declaration of Helsinki and following national and international guidelines. The study protocol was approved by the ethics committee of the Zhengzhou Traditional Chinese Medicine Traumatology Hospital. Written informed consent was obtained from all participants after a full explanation of the study. The experimental protocol was implemented in accordance with the approved guidelines.

### 2.2 Subjects

All subjects were members of Chinese Han population living in the Henan Province of China. The cases were recruited from Zhengzhou Traditional Chinese Medicine Traumatology Hospital, China. ONFH was diagnosed by examining osteonecrosis in anteroposterior and frog view X‐rays of both hips and/or magnetic resonance imaging. The ONFH patients with other direct trauma, chronic diseases (such as cardiovascular diseases, congenital diseases, human immunodeficiency virus infection, diabetes mellitus, renal dysfunction, and cancer), corticosteroids, alcohol, and familial hereditary diseases were excluded. Individuals in the control group had no ONFH disease. We recruited subjects without consideration of age and gender.

### 2.3 SNP selection and genotyping

We selected these SNPs on the basis of their allele frequencies, location, and disease relevance through public HapMap databases. All 11 SNPs had minor allele frequencies >5% in the 1,000 genome (http://www.internationalgenome.org/). Blood samples were collected in EDTA tubes and stored at −80°C after centrifugation at 17,528 *g* for 10 min. Genomic DNA was extracted from peripheral blood samples using a genomic DNA purification kit (GoldMag, Xi'an, China). We used NanoDrop 2000 (Thermo Scientific, Waltham, MA) to measure the DNA concentration. The primers for amplification and extension reactions were designed with Agena MassARRAY Assay Design 3.0 Software (Gabriel, Ziaugra, & Tabbaa, 2009). We used Agena MassARRAY RS1000 to perform the SNP genotyping with the agreement of the manufacturer, and we used Agena Typer 4.0 software for data management and analysis (Gabriel et al., 2009; Thomas et al., 2007).

### 2.4 Statistical analysis

Microsoft Excel (Microsoft, Redmond, WA) and SPSS Statistics (version 17.0, SPSS, Chicago, IL) were used for statistical analyses. All *p*‐values were two‐tailed, and *p* < 0.05 was considered to be statistically significant. SNP genotype frequencies in the case and control groups were calculated by chi‐squared test, and the Hardy–Weinberg equilibrium (HWE) values were used to check the genotype frequency of the control group. Unconditional logistic regression analysis was used to examine the odds ratios (ORs) and 95% CIs in order to assess the association between SNPs and ONFH (Bland & Altman, 2000). Four models (codominant, dominant, recessive, and log‐additive) were used to test the association between SNPs and ONFH (Sole, Guino, Valls, Iniesta, & Moreno, 2006). Finally, the Haploview software package (version 4.2) and SHEsis software platform (http://www.nhgg.org/analysis/) were used to estimate pairwise linkage disequilibrium, haplotype construction, and genetic association at polymorphism loci.

## 3 RESULT

### 3.1 Characteristics of the participants

This study involved 727 subjects, including 286 patients (173 males and 113 females; age at diagnosis: 41.83 ± 13.11 years) and 441 healthy controls (265 males and 176 females; age: 44.60 ± 11.55 years). The stroke cases and controls were matched by sex, but there was a significant difference in age between stroke cases and controls (*p *<* *0.05) (Table 1).

**Table 1:** General characteristics the of this study population

Variables	Cases (*n* = 286)	%	Controls (*n* = 441)	%	*p*‐value
Sex					
Male	173	60.50	265	60.10	>0.05
Female	113	39.50	176	39.90	
Age, year (mean ± *SD*)	41.83 ± 13.11		44.60 ± 11.55		<0.05

Note

*p*‐values were calculated from two‐sided chi‐squared test/Fisher's exact test.

*p *≤* *0.05 was statistically significant.

### 3.2 The associations between IL1R1 and IL1R2 SNPs and ONFH

Eleven SNPs in *IL1R1* and *IL1R2* were analyzed in this study. Allele frequencies and basic information for all SNPs are shown in Table 2. One SNP (rs12712127) was excluded for significant deviation from HWE (*p* < 0.05). We used the chi‐squared test to assess the risk of gene polymorphism in the allele model, while rs11674595 in *IL1R2* was significantly associated with increasing the ONFH risk (rs11674595, OR = 1.37, 95% CI = 1.07–1.75, *p *=* *0.012), the rs10490571 and rs3917225 in *IL1R1* gene were associated with an increased risk of ONFH (rs10490571, OR = 1.36, 95% CI = 1.04–1.77, *p *=* *0.025; rs3917225, OR = 1.35, 95% CI = 1.09–1.68, *p *=* *0.006), respectively.

**Table 2:** Allele frequencies in cases and controls and odds ratio estimates for ONFH risk

SNP	Gene(s)	Band	Alleles A/B	MAF		*p*‐HWE	OR (95% CI)	*p*‐value
				Case	Control			
rs11674595	*IL1R2*	2q11.2	C/T	0.267	0.211	0.250	1.37 (1.07–1.75)	**0.012** a
rs4851527	*IL1R2*	2q11.2	A/G	0.259	0.289	0.486	0.86 (0.68–1.09)	0.214
rs719250	*IL1R2*	2q11.2	T/G	0.333	0.317	0.912	1.07 (0.86–1.35)	0.533
rs3218896	*IL1R2*	2q11.2	C/T	0.173	0.150	0.351	1.18 (0.89–1.57)	0.252
rs3218977	*IL1R2*	2q11.2	G/A	0.263	0.251	1.000	1.07 (0.84–1.36)	0.592
rs2072472	*IL1R2*	2q11.2	G/A	0.208	0.200	0.766	1.05 (0.81–1.37)	0.696
rs10490571	*IL1R1*	2q12.1	T/C	0.215	0.168	0.061	1.35 (1.04–1.77)	**0.025** a
rs12712127	*IL1R1*	2q12.1	G/A	0.290	0.215	**0.000**	1.50 (1.17–1.91)	0.001
rs956730	*IL1R1*	2q12.1	A/G	0.248	0.250	0.016	0.99 (0.78–1.26)	0.940
rs3917225	*IL1R1*	2q12.1	T/C	0.419	0.348	0.074	1.35 (1.09–1.68)	**0.006** a
rs3917318	*IL1R1*	2q12.1	G/A	0.498	0.497	0.045	1.01 (0.82–1.24)	0.951

Note

SNP: single‐nucleotide polymorphism; MAF: minor allele frequency; HWE: Hardy–Weinberg equilibrium; OR: odds ratio; CI: confidence interval.

a In bold, *p* < 0.05 indicates statistical significance

In bold, *p*‐HWE < 0.05 be excluded.

### 3.3 Associations between genotype frequencies and osteonecrosis risk

As is shown in Table 3, we examined whether the minor allele for each SNP compared to the wild‐type allele represented a risk factor in the genetic model. Our analyses showed that the rs11674595 in *IL1R2* gene was associated with a 1.49‐fold increase the risk of ONFH in the codominant model (adjusted, OR = 1.49, 95% CI = 1.09–2.03, *p *=* *0.033 for the “C/T” genotype), 1.40‐fold increase the risk of ONFH in the dominant model (adjusted, OR = 1.50, 95% CI = 1.10–2.03, *p *=* *0.009 for the “C/T‐C/C” genotype), and 1.40‐fold increase the risk of ONFH in the log‐additive model (adjusted OR = 1.40, 95% CI = 1.08–1.81, *p *=* *0.006), respectively. The rs10490571 in *IL1R1* gene was associated with a 1.49‐fold increase the risk of ONFH in the codominant model (adjusted, OR = 1.69, 95% CI = 1.22–2.34, *p *=* *0.006 for the “C/T” genotype), 1.57‐fold increase the risk of ONFH in the dominant model (adjusted, OR = 1.57, 95% CI = 1.15–2.16, *p *=* *0.005 for the “C/T‐T/T” genotype), and 1.34‐fold increase the risk of ONFH in the log‐additive model (adjusted OR = 1.34, 95% CI = 1.03–1.75, *p *=* *0.031), respectively. The rs3917225 in *IL1R1* gene was associated with a 1.50‐fold and 1.65‐fold increase the risk of ONFH in the codominant model (adjusted, OR = 1.50, 95% CI = 1.07–2.09, *p *=* *0.022 for the “A/G” genotype; OR = 1.65, 95% CI = 1.06–2.58, *p *=* *0.022 for the “G/G” genotype), respectively. The rs3917225 was associated with a 1.54‐fold increase the risk of ONFH in the dominant model (adjusted, OR = 1.57, 95% CI = 1.13–2.10, *p *=* *0.006 for the “A/G‐G/G” genotype) and 1.32‐fold increase the risk of ONFH in the log‐additive model (adjusted OR = 1.32, 95% CI = 1.07–1.64, *p *=* *0.009), respectively.

**Table 3:** Relationships between *IL1R2* and *IL1R1*polymorphism and ONFH risk

SNP	Model	Genotype	Control	Case	Before adjusted		After adjusted		AIC	BIC
					OR (95% CI)	*p* avalue	OR (95% CI)	*p* bvalue		
rs11674595	Codominant	T/T	269 (61.3%)	147 (51.4%)	1	**0.03**	1	**0.033**	971.5	985.3
		C/T	155 (35.3%)	125 (43.7%)	**1.48 (1.08–2.01)**		**1.49 (1.09–2.03)**			
		C/C	15 (3.4%)	14 (4.9%)	1.71 (0.80–3.64)		1.60 (0.75–3.42)			
	Dominant	T/T	269 (61.3%)	147 (51.4%)	1	**0.009**	1	**0.009**	969.6	978.8
		C/T‐C/C	170 (38.7%)	139 (48.6%)	**1.50 (1.11‐2.02)**		**1.50 (1.10–2.03)**			
	Recessive	T/T‐C/T	424 (96.6%)	272 (95.1%)	1	0.33	1	0.420	975.6	984.7
		C/C	15 (3.4%)	14 (4.9%)	1.45 (0.69–3.06)		1.36 (0.64–2.88)			
	Log‐additive	–	–	–	**1.41 (1.09–1.83)**	**0.01**	**1.40 (1.08–1.81)**	**0.012**	969.8	979
rs10490571	Codominant	C/C	310 (70.5%)	172 (60.1%)	1	**0.005**	1	**0.006**	969.1	982.9
		C/T	112 (25.4%)	105 (36.7%)	**1.69 (1.22–2.34)**		**1.69 (1.22–2.34)**			
		T/T	18 (4.1%)	9 (3.1%)	0.90 (0.40–2.05)		0.88 (0.39–2.01)			
	Dominant	C/C	310 (70.5%)	172 (60.1%)	1	**0.004**	1	**0.005**	969.3	978.5
		C/T‐T/T	130 (29.6%)	114 (39.9%)	**1.58 (1.16–2.16)**		**1.57 (1.15–2.16)**			
	Recessive	C/C‐C/T	422 (95.9%)	277 (96.8%)	1	0.51	1	0.470	977.1	986.3
		T/T	18 (4.1%)	9 (3.1%)	0.76 (0.34–1.72)		0.74 (0.33–1.69)			
	Log‐additive	–	–	–	**1.35 (1.03–1.76)**	**0.027**	**1.34 (1.03–1.75)**	**0.031**	972.6	981.8
rs3917225	Codominant	A/A	196 (44.5%)	98 (34.4%)	1	**0.02**	1	**0.022**	969.9	983.6
		A/G	182 (41.4%)	135 (47.4%)	**1.48 (1.07–2.06)**		**1.50 (1.07–2.09)**			
		G/G	62 (14.1%)	52 (18.2%)	**1.68 (1.08–2.61)**		**1.65 (1.06–2.58)**			
	Dominant	A/A	196 (44.5%)	98 (34.4%)	1	**0.006**	1	**0.007**	968.2	977.4
		A/G‐G/G	244 (55.5%)	187 (65.6%)	**1.53 (1.13–2.09)**		**1.54 (1.13–2.10)**			
	Recessive	A/A‐A/G	378 (85.9%)	233 (81.8%)	1	0.14	1	0.170	973.4	982.6
		G/G	62 (14.1%)	52 (18.2%)	1.36 (0.91–2.04)		1.33 (0.89–2.00)			
	Log‐additive	–	–	–	**1.33 (1.08–1.64)**	**0.008**	**1.32 (1.07–1.64)**	**0.009**	968.6	977.8

Note

SNP: single‐nucleotide polymorphism; OR: odds ratio; 95% CI: 95% confidence interval.

a *p*‐values were calculated from unconditional logistic regression analysis.

b *p*‐values were calculated by unconditional logistic regression analysis with adjustments for age and gender.

The bold values and *p* ≤ 0.05 indicate statistical significance.

### 3.4 Associations between haplotype analyses and ONFH risk

**Figure 1:**
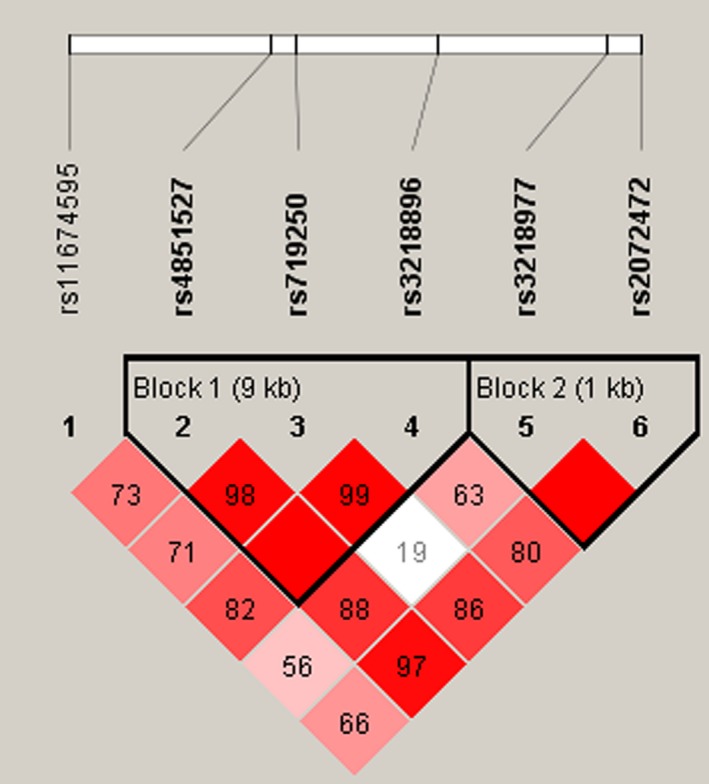
Linkage disequilibrium plots containing four SNPs from *IL1‐R2*

Linkage disequilibrium and haplotype analyses of the SNPs in the case and control samples were further studied. Haplotype analysis revealed two blocks in the *IL1R2* gene (Figure 1). Although the three SNPs (rs4851527, rs719250, and rs3218896) and the two SNPs (rs3218977 and rs2072472) in the *IL1R2* gene have showed strong linkage, but the result for the *IL1R2* haplotype was not found to be associated with a risk of ONFH, because the *p*‐value has no statistical difference (Table 4). In addition, we have not found any association between *IL1R1* haplotype and the risk of ONFH.

**Table 4:** Haplotype analysis results in this study

Block	SNPs	Haplotype	Freq	Before adjusted		After adjusted	
				OR (95% CI)	*p*‐value	OR (95% CI)	*p* ^a^‐value
Block 1	rs4851527/rs719250/rs3218896	GCT	0.4011	1	–	1	–
		ACT	0.2749	0.88 (0.67–1.14)	0.33	0.87 (0.67–1.14)	0.32
		GTT	0.1638	0.93 (0.68–1.27)	0.65	0.93 (0.68–1.28)	0.68
		GTC	0.1583	1.13 (0.82–1.54)	0.45	1.13 (0.82–1.55)	0.45
Block 2	rs3218977/rs2072472	AA	0.5411	1	–	1	–
		GA	0.2555	1.09 (0.85–1.41)	0.51	1.08 (0.84–1.40)	0.55
		AG	0.2035	1.09 (0.82–1.43)	0.56	1.07 (0.81–1.42)	0.63

Note

SNP: single‐nucleotide polymorphism; OR: odds ratio; CI: confidence interval; *p*a: Adjusted by gender and age.

## 4 DISCUSSION

Genetic studies have provided insight into numerous diseases, including ONFH. In the present case–control study, we investigated the associations between 11 SNPs in *IL1R1* and *IL1R2* gene risk of ONFH. We demonstrated that *IL1R2* and *IL1R1* genetic polymorphisms were associated with ONFH risk in Chinese Han population. Our results indicate that the rs11674595 (in the *IL1R2*), the rs10490571, and rs3917225 (in the *IL1R1*) were associated with an increased risk of ONFH. These results suggest that polymorphisms in *IL1R2* and *IL1R1* genes may play an important role in the risk of ONFH in the Han Chinese population.

The biological activity of the multifunctional cytokine *IL‐1* is mediated by its receptors (Lukens, Gross, & Thirumala‐Devi, 2012). The *IL1R1* and *IL1R2* genes are cytokine receptors that belong to the *IL‐1* receptor family, which is an important mediator involved in many cytokine‐induced responses (Sims & Dower, 1994). *IL1R1* (on chromosome 2q12) is an important mediator involved in many cytokine‐induced immune and inflammatory responses (Dinarello, 1998). Recently, some studies revealed that the expression of IL1R1 was observably increased in several bone disease. For example, Latiano et al. (2013) reported that the rs13015714 and rs2058660 in *IL1R1* could increase the risk of IBD; Kouhia et al. (2010) indicated that four SNPs (rs1465325, rs956730, rs3917225, and rs2287047) in the *IL1R1* gene provided evidence for association with hand osteoarthritis. Another study involving the association between five SNPs polymorphisms in IL1R1 (rs10490571, rs12712127, rs956730, rs3917225, and rs3917318) and osteoarthritis risk, the result found that rs3917225 in IL1R1 was associated with increasing the risk of knee OA (Na et al., 2017; Smith et al., 2004). However, we have not found any evidence for the role of heredity between *IL1R1* and ONFH susceptibility in previous study. Therefore, our study fully discusses the relationship between *IL1R1* and ONFH risk. In our case–control study, we found that rs10490571 and rs3917225 were associated with an increased risk of ONFH.

*IL1R2* (on 2q11.2) is a molecular decoy that traps IL‐1β and does not initiate subsequent signaling events, thereby suppressing an inflammatory response. Many studies reported that the *IL1R2* as a risk factor in some disease, such as IgA nephropathy (Xie et al., 2017). The other study reported that epithelial *IL1R2* acted as a homeostatic regulator during remission of ulcerative colitis (Mora‐Buch et al., 2016), and another suggested that *IL1R2* was an important regulator of arthritis by acting specifically on macrophages as a decoy receptor for *IL‐1* (Shimizu et al., 2015). However, previous studies based on the *IL1R2* gene polymorphisms were rare, until now, there has no study reported the association between *IL1R2* and the ONFH risk. For this study, the rs11674595 in *IL1R2* showed an increased risk of ONFH. Hence, *IL1R2* gene may play an important function in affecting ONFH.

To sum up, we provide new evidence for the association between *IL1R1* and *IL1R2* variant and ONFH risk in Han Chinese population for the first time, which may provide new data to facilitate earlier diagnosis and promote early prevention, and shed light on the new candidate genes and new ideas for the study. Nevertheless, there are limitations that need to be noticed. Our current research is fundamental, and further functional studies and larger population‐based prospective studies are required in order to understand the genetic factors underlying ONFH.

## CONFLICTS OF INTEREST

None.
